# EPIStress: A multimodal dataset of Physiological signals to measure cognitive stress in epilepsy patients

**DOI:** 10.1038/s41597-025-06328-3

**Published:** 2025-11-25

**Authors:** Sidratul Moontaha, Constanze Cavalier, Birgitta Esser, Arthur Jordan, Ines Goebel, Christoph Anders, Afsana Mimi, Björn Krüger, Rainer Surges, Bert Arnrich

**Affiliations:** 1 University of Potsdam, Digital Engineering Faculty, Digital Health - Connected Healthcare of the Hasso Plattner Institute, Potsdam, 14482 Germany; 2 Department of Epileptology, University Hospital Bonn, Bonn, 53127 Germany; 3 Department of Computer Science, University of Bonn, Bonn, Germany

**Keywords:** Biomedical engineering, Quality of life

## Abstract

Epilepsy patients commonly report stress as a frequent seizure trigger; however, the objective seizure-stress relationship is unclear due to self-report biases and difficulty in objective quantification of stress. This work presents a dataset from twenty epilepsy patients undergoing cognitive stress elicitation protocols, participating in laboratory experiments with computer-based tasks at predefined difficulty levels, and in situational experiments by independently choosing tasks with at least two difficulty levels. Physiological signals from wearable electroencephalography, photoplethysmography, acceleration, electrodermal activity, and temperature sensors were recorded. The task-related perceived cognitive stress was collected using two 5-point Likert scales of self-reported mental workload and stress, contrasted by a pairwise NASA-TLX questionnaire. Additionally, the dataset includes a patient-reported list of seizure-provoking and -inhibiting factors. Results illustrated individual and heterogeneous responses to cognitive tasks, with some modalities yielding statistically significant features, while others demonstrated expected directional trends. The findings support the validity and suitability of the proposed dataset for cognitive stress detection and the potential to map seizure-related factors to cognitive stress events.

## Background & Summary

Approximately 40 to 70 individuals per 100,000 people worldwide, and about 55 to 60 per 100,000 in Europe, have epilepsy, and stress is widely recognized as the most common trigger for seizures for these patients^1^. Many patients report subjective experiences such as auras (occurring seconds to minutes before), prodromes (occurring several minutes to two days before), or precipitant factors (occurring earlier than 2 days before) a seizure occurrence. These early warning signs of manifesting seizures—ranging from sensory and behavioral to cognitive or emotional changes—are highly subjective. However, successful self-control of seizures was reported by applying behavioral strategies such as preparing for consequences, cognitive strategies such as diverting focus, or emotional strategies such as relaxing and positive thinking^1^.

Although self-identification of warning signs offers a promising avenue for anticipating seizures^2,3^, relying solely on subjective alert symptoms is often unreliable, since the sensitivity of perception can vary significantly across time. Therefore, the objective relationship between the mental states of stress, cognitive load, and emotions to seizures must be investigated despite various methodological challenges. The growing interest in developing external support tools based on physiological signals to aid seizure detection, prediction^4^, and forecasting underlines this need. Seizure detection and prediction systems typically trigger an alarm when detecting the pre-ictal period, a phase revealing the earliest sign of seizure activity. In contrast, forecasting systems consistently use probabilistic approaches to provide seizure risk assessments to patients^5–7^. Consequently, developing a system that records physiological data and subjective perceptions of mental state and seizure triggers is essential for supporting personalized seizure risk profiling.

As such, researchers from the epilepsy community are developing seizure forecasting systems with available datasets like the *EPILEPSIAE* database^4^ or their own collected data^5,8^. Although these datasets often include physiological signals and seizure diaries, and despite the clinical evidence linking mental state to seizure risks, they typically overlook mental health factors. As a result, the comprehensive seizure profiling analysis of such datasets is limited. In contrast, researchers from the cognitive stress domain are working on building robust cognitive load detection tools from the datasets available, as listed in Table 1. However, the existing research works on cognitive stress detection are limited to either i) using existing datasets collected primarily from healthy participants, ii) using clinical, invasive, or obtrusive physiological measures, iii) lacking context information such as the hospital setup for patients, or iv) a general lack of behavioral data.

**Table 1:** The abbreviations used in the table: RESP: respiration, BVP: Blood Volume Pulse, EDA: Electrodermal Activity, TEMP: Skin Temperature, ACC: Acceleration, HR: Heart Rate, IBI: Inter-Beat Interval, PPG: Photoplethysmogram, and RR: beat-to-beat intervals.

Publication	Scenario	Modality (Questionnaires)	Study Environment	Cohort	Wearables	Participants
Office Tasks 2019^33^	Stress	Cameras, Visual EDA, HR, Breathing Rate (Standard)	Lab	Primarily Students	2	63
WESAD^34^	Stress	BVP, ECG, EDA, EMG, RESP, ST and ACC	Lab	Graduate Students	2	15
Snake and CogLoad^35^	Cognitive Load	ACC, EDA, TEMP and RR	Lab	Healthy Participants	1	23 (Snake) + 23 (Load)
Deskbound Research^36^	Cognitive Load	Cameras (Standard)	In Situ	Faculty, postdoctoral, and doctoral researchers	0	10
COG-BCI^37^	Cognitive Load	EEG (Standard)	Lab	Primarily Students	0	29
A test-retest dataset^38^	Mental State	EEG (Standard)	Lab	Healthy Participants	0	60
Stress Dataset^39^	Stress	EDA, HR, ST, ACC, IBI, BVP (Non standard)	In Situ	Nurses	1	15
AKTIVES^40^	Stress	BVP, EDA, ST, Facial Expressions, ACC (Non standard)	Lab	Children with special needs	1	25
K-EmoPhone^41^	Mental State	ACC, HR, ST, EDA, IBI, and phone sensors (Standard)	In Situ	Students	1 + phone	77
Universe^10^	Mental State	ACC, HR, ST, EDA, EEG, PPG, and Log Data (Standard)	Both	Primarily Students and Researchers	2	24
MOCAS^42^	Cognitive Load	EEG, PPG, GSR, HR, facial and eye features, and mouse movement	Lab	Students and faculty/staff	4	30
**EPIStress (Proposed)**	**Mental State**	**ACC, HR, ST, EDA, EEG, PPG, and Log Data** (Standard)	**Both**	**Epilepsy Patients**	**2**	**20**

The state-of-the-art datasets in the domain of cognitive stress with the proposed dataset in this paper. The significant difference lies in the type of chosen cohort. The study environment is primarily chosen in laboratory settings other than the current one and the previous one published by the authors. The non-invasive multi-modality is also ensured for the proposed dataset.

The proposed dataset is created to bridge this gap in the literature by identifying patterns of cognitive stress in epilepsy patients, possibly differentiating between individuals or states (e.g., stressed vs. not stressed), characterizing individual differences with the control group, and understanding stress-trigger relationships. The dataset was collected during a laboratory and an in-situ experiment consisting of three main components: i) physiological sensor data from wearable electroencephalography (EEG), photoplethysmography (PPG), acceleration (ACC), electrodermal activity (EDA), and temperature (Temp) sensors, ii) self-reported cognitive stress, emotion and mental workload levels, and iii) a list of individualized seizure triggers.

The dataset is available in four types:

1. the original, **raw** physiological data with patient-reported task activities, stress and mental workload levels, useful for unsupervised algorithms
2. the synchronized and task-wise segmented **labeled** data, ready to use for deep learning models
3. the basic **preprocessed** data to investigate deep learning and machine learning algorithms
4. **feature** set of different window sizes, to understand the initial task-specific feature variations

Additionally, the response to the seizure trigger questionnaire, the additional information about the experiments as notes, the timestamps, log files, and answers and durations to the task-specific responses are provided, offering a potential source for exploratory data analysis to discover multiple research questions.

**Fig. 1:**
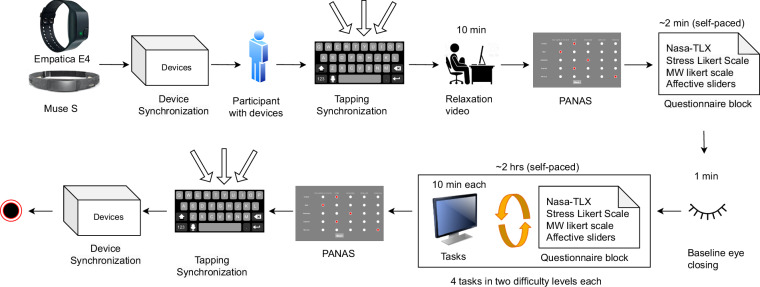
Experimental protocol followed in the laboratory: two wearable devices were used for data collection after performing two levels of synchronization. Starting with a relaxation video, participants were given four tasks, each with two difficulty levels, for a total duration of ten minutes each. Participants reported subjective scores on four questionnaires—pairwise NASA-Task Load Index (NASA-TLX), 5-point Likert Scales for mental workload, 5-point Likert Scales for stress, and affective sliders—between each task, and the experiment ended with another synchronization protocol. The Positive and Negative Affect (PANAS) questionnaire was reported at the beginning and end of the experiment.

**Fig. 2:**
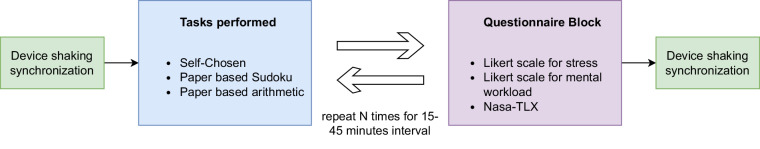
Experimental protocol followed in the situ set up: the Muse S headband and Empatica E4 devices were used after performing one level of synchronization, participants performed self-chosen tasks and answered the questionnaire blocks every 15 to 45 minutes for 2 hours. The experiment ended with another synchronization step.

The assays used in the dataset and the type of data collected are described in the *Methods* section and visually depicted in Figs. 1 and 2. The *Data Records* section describes the four individual types of the provided data mentioned above and additional data. With the primary objective of detecting cognitive stress in epilepsy patients, the dataset can be used to answer research questions in the area of generalized objective stress and subjective trigger analysis, to compare cognitive responses and feature distributions with control groups, and to provide additional contextual information to seizure forecasting systems.

## Methods

### Patient Recruitment

The ethics committee at the Medical Faculty of the *Rheinische Friedrich-Wilhelms-University Bonn* provided the ethical approval for this study under the reference number *096/23-EP*. An initial patient screening was conducted at the *Department of Epileptology of the University Hospital Bonn*, where all data collections took place. Patients eligible for inclusion had to be adults aged 18 years or older, diagnosed with focal or generalized epilepsy for at least two years already, and who had experienced at least one seizure within the last six months. Additional criteria required the participants to have a basic proficiency in using desktop computers and smartphones, as well as a fluency in German or English.

Exclusion criteria excluded individuals under 18 years of age, those with an epilepsy diagnosis less than two years ago, or those who did not have a (recorded) seizure in the last six months. Patients were also excluded if they were unable to operate desktop computers or smartphones, were not fluent in either German or English, had a history of learning disabilities, neurosurgery, psychiatric conditions, or severe mental health issues related to mental workload or stress. A study nurse provided the eligible patients with a study information sheet and gave a verbal explanation of the study. Patients provided their written consent for participation and the publication of anonymized data. Patients were allowed to ask questions throughout the data collection until they were satisfied, and informed they could quit their participation before, after, or even during an ongoing experiment without any consequences or the need to provide any explanation. They received remuneration for participation.

A total of 20 patients (8 males and 12 females) participated in the study, with an age range of 19 to 55 years, and a mean age of 30.7 (±9.8%) years. Additionally, patients had 10 to 15 years of formal education. In terms of anti-seizure medication (ASM), 30% of the patients were receiving mono-therapy, whereas the remaining 70% were receiving poly-therapy during the period of data collection. Data were collected from 13 inpatients and 7 outpatients, while 5 of the inpatients were at the hospital for complex treatments (*Komplexbehandlung*: https://klassifikationen.bfarm.de/ops/kode-suche/htmlops2025/block-8-97...8-98.htm). As classified by Scheffer *et al*.^9^, the patients have focal epilepsy of structural (40%), unknown (30%), and immune (10%) etiology, and generalized epilepsy with genetic etiology (15%). In half of the patient population, neuro-imaging demonstrated structural cerebral lesions.

### Experimental Paradigm

An experimental design was developed to curate data from the patients in a designated room, e.g., laboratory experiment or in-situ setup at the Department of Epileptology of the University Hospital Bonn, distributed between different days and times throughout the year 2024. Therefore, the dataset includes physiological and questionnaire data from varying days and times, following two experimental protocols: laboratory setup and the in-situ setup.

#### Laboratory Setup

In the laboratory setup, the patients were asked to sit comfortably on a chair in front of a laptop screen (Lenovo laptop with 16 GB of RAM, an Intel Core i7 processor, running Ubuntu 20.04 LTS) on a desk with a keyboard and mouse. The room was temperature-controlled and semi-soundproofed from external noises. One or two experimenters were allowed to be in the room with the patient. The patients were allowed to take breaks to go to the toilet or walk during the experiments, which happened only in two instances. Even minimal external distractions during the sessions were noted. Participants were allowed to drink water during their data recording sessions. After the consent form was signed, the participants were once again briefed about the experiments by showing a fast-forward video of the experiment, with pauses to give verbal explanations or answer any questions from the participants. The video showed the screen recording of the PsychoPy application (v2022.2.1, developed in Python 3.8), which is a automated tool to guide the participant in performing the tasks during the experiment. Keeping the original strengths—modular task loader and multiple device-synchronization protocols—of the previous version^10^, the current version of the Psychopy tool added upgrades like safe-quit: abort any time without corrupting data and bilingual UI: startup drop-down to choose study language between English or German, with all text pulled from language tables. The project remains open-source to clone it at: https://github.com/HPI-CH/EPIStress/tree/main/Psychopy. The seizure trigger questionnaire was subsequently explained to the participants to fill it out at own pace. Additional questions on age, gender, and educational qualifications were asked before starting the experiment.

The data collection protocol in the laboratory is illustrated in Fig. 1. To start the procedure, the experimenter started the Muse S and Empatica E4 devices (see details on subsection *Physiological Data Acquisition*) and opened the respective applications. The devices were then taken and shaken very vigorously to have a high amplitude in the acceleration peak to synchronize the data. Details are provided in the authors’ previous publication^10^. The experimenter placed the Muse S device on the forehead and the Empatica E4 device on the wrist of the participant. The headband had to be adjusted according to the head size of the participant. A proper fit of the Muse S headband was ensured by checking the Horse Shoe Indicator (HSI) shown on the Mind Monitor application (https://mind-monitor.com/), where solid ovals, outlines, and empty spaces represent good connections, poor connections, and no connections, respectively. A good connection of the Empatica E4 device was ensured by looking at the E4 real-time app, which shows “device not on the wrist” in case of poor contact with the skin. Moreover, the heart rate computed by the application is visible only after the connection is properly established. The E4 device and the real-time app are no longer available following the release of EmbracePlus (https://www.empatica.com/research/e4-sunset/). Once proper device fit had been established, the participant put on the headphones, and the PsychoPy application was started. Within this computerized application, the participants were instructed to perform another synchronization step, synchronizing between PsychoPy and the E4 device. Consequently, the participant had to tap the space bar swiftly four times on the keyboard from a very high amplitude, using their non-dominant hand in which the E4 device was worn. Participants were then asked to reduce their body movement, especially of the non-dominant hand and of the head. The PsychoPy application guided the participant through the experiment, showing a relaxation video for 10 minutes at the beginning, without further interruption from the experimenter, who sat opposite the participant. This procedure was aimed at reducing stress prior to coming to the lab or during the lab environment. The current affective states were subjectively reported by the participants answering the Positive and Negative Affect (PANAS) questionnaire^11^. Next, a set of questionnaires appeared consisting of two 5-point Likert Scales for mental workload and stress^12^, followed by the pairwise NASA-TLX questionnaire^13^, and affective sliders^14^ as mentioned in detail in subsection (*Task Questionnaires*). After answering the questionnaires, the participants performed an eye-closing session for one minute. Afterwards, the participants were asked to perform four tasks in two difficulty levels, resulting in a total of eight different tasks—each performed for 10 minutes—in random order. The task descriptions follow:

- **Sudoku** challenges players to logically fill a 9 × 9 grid so that every row, column, and 3 × 3 subgrid includes the digits 1 through 9 exactly once, without repetition. A digital version of the Sudoku application was presented^15^, allowing participants to perform only easy and hard difficulty levels, among others. The participants can play multiple times if required. The application closed automatically after ten minutes.
- **N-back task** is a cognitive memory task commonly used to measure working memory and attention in which participants are presented with a continuous stream of stimuli—such as letters, numbers, sounds, or images—and must indicate when the current stimulus matches the one that appeared *n* steps earlier in the sequence^16^. In the setup utilized, the participants observed a sequence of colored rectangles of six colors: red (), magenta (), cyan (), green (), white (), and blue (). Depending on the difficulty level, they had to match it with the square one or two steps earlier, referring to 1-back (easy) and 2-back (hard) levels, respectively. Each block appeared for 2 seconds, during which participants could press the space bar to indicate observing a correct match.
- **Mental arithmetic task:** while doing mental arithmetic tasks, participants completed calculations without external aids such as papers or calculators. Solving math problems entirely in the mind can trigger a significant cognitive load. The hard level involved multi-digit multiplication and division using a range of operands between −100 and 100. In the interest of time and cognitive load, rounding to the nearest integer was considered a correct answer. The easy level focused on adding and subtracting within the same operand range, with or without carry numbers.
- **Stroop task:** evaluates executive functioning and cognitive control abilities by assessing an individual’s selective attention and processing speed^17^. A series of colored words appeared on the screen in varying font colors, which either matched or conflicted with the name of the color. In this paper, four colors were used: red, blue, yellow, and green. The participants had to identify and type the first letter of the font color, ignoring the word itself. Since the names of the colors were in German and both green (grün) and yellow (gelb) have the same initials in German, the participants were asked to use y for the color yellow, a potential added cognitive load for non-native English speakers. The task difficulty was controlled through time constraints. The maximum response times given were 1.5 and 5.5 seconds for hard and easy conditions, respectively.

The participants underwent a trial session of 45 seconds each for the N-back, Stroop, and arithmetic tasks. Answers to these tasks and reaction times were systematically recorded during each experiment. After each task with a defined difficulty level, the block of the questionnaire set was repeated throughout the experiment. After each task and questionnaire block, a prompt appeared on the screen, asking participants whether they were willing to proceed to the next task, allowing for early-stopping and breaks. The experiment ended with either the unwillingness to continue or the participants finishing all eight tasks. Finally, another PANAS questionnaire was answered to detect the overall changes in the affective state before or after the experimental session. The experimenter helped the participant again with the space bar tapping synchronization, just as in the beginning. The devices were taken off and synchronized with the same device-shaking protocol. The overall duration for each experiment and the number of tasks performed per person are depicted in the table 3.

#### In Situ Setup

Before starting data collection in the situ setup, participants were briefed on the study and the device usage in a designated room at the hospital, with adequate time allocated for questions. Depending on the order of the session, i.e., if not completed earlier, the seizure trigger questionnaire was filled out.

The data collection protocol is depicted in Fig. 2. The experimenter connected the devices (see details on subsection *Physiological Data Acquisition*) with the respective applications, synchronized the devices, and fitted the devices to the participant. Participants were then allowed to return to their respective locations, such as their rooms, the hospital lobby, the designated waiting area for patients, or the walking zone outside of the building but within the hospital premises. They were instructed to collect data for approximately two hours by themselves performing high and low cognitive stress tasks by switching between tasks every 15 to 45 minutes. After every task they were suppose to complete a pen-and-paper-based questionnaire, reporting task details, breaks, and additional comments, along with the three questionnaires NASA-TLX and the Likert scales for mental workload and stress. Participants carried the phone with the application on it with them at all times, and the experimenter checked the device connection at least every 30-45 minutes, to see if data were collected as intended.

After two hours, the devices were returned, and the participants completed any missing questionnaire data. The experimenters finished the session by following the final synchronization protocol with the devices. The duration and number of tasks performed per participant are reflected in Table 3.

The authors categorized self-chosen tasks into five categories based on the several tasks reported by the participants: **watching content on their smartphone,** **reading,** **walking and other low-effort physical activities,** **playing Sudoku**, and **arithmetic tasks**. Watching content on a smartphone included watching movies, videos, series, or sports and using the phone for social media or messaging, as reported by the patients. Reading referred to reading English comics and other unspecified materials. Walking and physical activities included walking, climbing stairs, going to the toilet, visiting the cafeteria, strolling while reading, interacting with the doctor, and attending therapy sessions. Since it was challenging to find mentally demanding tasks for inpatients or outpatients during their hospital stays, participants were given pen-and-paper-based Sudoku and arithmetic tasks with the freedom to choose independently between these. The Sudoku options included 4 × 4, 6 × 6, and 9 × 9 grids. The arithmetic task included addition, subtraction, division, and multiplication of operands ranging from  − 100 to 100, potentially involving carry numbers. As depicted in Fig. 7, reading and watching content on smartphones were frequently chosen as low-load tasks, whereas arithmetic tasks and playing Sudoku were chosen as high-workload tasks (by the participants). Walking and physical activities had mixed ratings. In one instance, the participant was given a laptop with an internet connection to perform a relaxation activity.

### Physiological Data Acquisition

Physiological signals were continuously recorded throughout the experiment to obtain objective markers of stress and workload. Table 2 lists two recording devices for collecting physiological data from the participants: the Muse S headband and the Empatica E4 wristband. The Muse S headband records Electroencephalography (EEG) signals from five sensor locations, AF7, AF8, TP9, TP10, and FpZ, according to the 10/20 international system. It also records Accelerometer (ACC_Muse) and Gyroscope (Gyro) data. Though it has a Photoplethysmography (PPG) sensor, the raw PPG data is not accessible with the third-party app Mind Monitor, which gives only raw EEG, ACC, and Gyro data and additional calculated power features from the EEG data. Mind Monitor saves data from all sensors at a sampling rate of 256 Hz, the sampling frequency of the EEG sensor, whereas the actual sampling frequency of the ACC sensor is 52 Hz. As mentioned in the previous section (*Experimental Paradigm*), the app shows a Horse Shoe Indicator (HSI), depicting the connection stability. Recorded data is stored locally in a.csv format via the app.

**Table 2:** Data Recording devices and the recorded modalities with their respective frequencies in Hz.

Recording Device	Modality	Abbreviation	Frequency in Hz	Unit
Muse S	Electroencephalography	EEG	256	*μ* V
	Accelerometer	ACC_Muse	256	ms^−2^
	Gyroscope	Gyro	256	^°^/s
Empatica E4	Electrodermal activity	EDA	4	*μ* S
	Skin temperature	ST	4	Celsius
	Accelerometer	ACC_E4	32	ms^−2^
	Inter-beat interval	IBI	64	s
	Blood volume pulse	BVP	64	bpm

**Table 3:** For each patient ID, total duration of recording in minutes, number of tasks (#Task) performed in the lab and in situ, and availability of trigger questionnaire are mentioned.

ID	Total Duration (min)	Lab (#Tasks) Pre-defined		In Situ (#Tasks) Stress score		Trigger Questionnaire
		Easy	Hard	Easy	Hard	
ES304	120	3	3	—	—	No
ES491	240	4	4	2	2	Yes
ES474	110	1	4	—	—	Yes
ES472	180	3	1	2	2	Yes
ES452	180	2	3	1	3	Yes
ES415	120	3	4	—	—	Yes
ES446	240	4	4	2	3	Yes
ES437	240	4	4	1	2	Yes
ES404	240	4	4	1	4	Yes
ES375	120	3	3	—	—	Yes
ES362	105	4	2	—	—	Yes
ES313	240	4	4	0	5	Yes
ES260	260	4	4	2	2	Yes
ES257	240	4	4	2	2	Yes
ES247	120	—	—	0	4	Yes
ES208	240	4	4	0	3	Yes
ES228	120	4	3	—	—	Yes
ES141	225	4	2	1	2	Yes
ES140	120	4	4	—	—	Yes
ES134	120	—	—	1	4	Yes

Total duration of recordings: ~116.50 hours; Muse: ~57 hours (Lab: ~33 hours, In Situ: ~24 hours), E4: ~59:00 hours (Lab: ~33 hours, In Situ: ~25 h). Number of tasks performed number of tasks: Lab 125, In Situ 53. The tasks are categorized into Easy and Hard for the Lab, with predefined labels, and for Situ, with Stress scores.

The Empatica E4 records Photoplethysmography (PPG), Electrodermal Activity (EDA), Temperature (TEMP), and Acceleration (ACC_E4) data at a sampling rate of 64 Hz, 4 Hz, 4 Hz, and 32 Hz, respectively. The E4 real-time app uploads the collected data to the designated server and is available in .csv format for download from the same source. Figure 1 illustrates both devices. Additionally, two Google Pixel phones (Pixel 3a; Android 12) were used with both the Mind Monitor and the E4 real-time app installed, and a specific folder for each participant had been created.

In the lab setup, two phones were used simultaneously, each running a single app to minimize the risk of data loss caused by apps being sent to the background, which happened only once during the test sessions. However, during the in situ data collection, participants were given only one phone to reduce the inconvenience of carrying two phones. Consequently, the Empatica E4 real-time app operated in the background, and the Mind Monitor app ran on the screen.

To present the experimental paradigm with the PsychoPy application, the Lenovo laptop was used. A designated folder for each participant was created, where the Psychopy logging functionality saved a .log file and a .csv file with the start- and end-timestamps of synchronization steps, of each task, of each questionnaire from the questionnaire set mentioned in section *Task Questionnaires*, and all the answers and durations of the answers available from the tasks. The PsychoPy application also provided two .csv files with the results from the two arithmetic tasks performed. The data from PsychoPy, along with the sensor data from the respective application and phone, were saved in each participant’s folder on the laptop.

### Behavioral Data Acquisition

Throughout the experiments, self-reported behavioral data were collected in the lab and in situ using standardize questionnaires to capture perceived mental states relevant to stress, workload, emotion and vulnerability to seizures. These instruments complement the physiological recordings to contextualize the individual perceptions in response to the experimental tasks.

#### Seizure-trigger Questionnaire

Patients completed a self-reported seizure trigger questionnaire with 66 seizure-related factors provided in German and English, adapted from Puteikis *et al*.^18^. As mentioned in Table 4, the factors include, among others, cognitive processes (e.g., positive or negative thoughts, focused thinking, mental calculation), daily activities (e.g. speaking, writing, working), auditory stimuli (e.g., unexpected loud sounds, sounds of a specific frequency, listening to a conversation). They identified provocative factors (*Anfall auslösend*) experienced before a seizure and inhibitors (*Anfall hemmend*) they avoid to try to prevent seizures. Additionally, they could also mention additional factors not listed in the items mentioned. Incorporating this data is particularly valuable in understanding inter-individual variability to support the broader goal of linking stress markers to seizure susceptibility.

**Table 4:** Group of items of potential seizure-related factors adapted from^18^, each item can be marked as a seizure-provoking factor or a seizure inhibitor.

Group of potentially seizure-related factors	Items in the group
Cognitive Processes & Thoughts	A specific memory, A specific thought, Positive thoughts, Negative thoughts, Other thoughts, Déjá vu, Focused thinking, Mental calculation, Written calculation, Decision-making, Problem-solving
Work & Daily Activities	Speaking, Writing, Reading, Working, Working with the computer
Creative & Leisure Activities	Computer games, Painting, Singing
Physical Activities & Exercise	Playing table games, Exercising, Dancing, Sexual activities, Complex movements
Substances & Stimulants	Alcohol, Caffeine, Smoking, Psychoactive substances (unspecified)
Sensory Perception & Stimuli	Pleasant gustatory stimuli, Unpleasant gustatory stimuli, Neutral gustatory stimuli, Pleasant smells, Unpleasant smells, Neutral smells, A specific taste/smell
Visual & Light Stimuli	Flickering lights, Single lights, Transition from darkness to light, Darkness, Eye-closing, Striped patterns, TV, a specific image/object/form/face
Auditory Stimuli	Unexpected loud sound, Music, Sounds of a specific frequency, Listening to a conversation, a specific voice, Phone sounds, Playing a musical instrument, A specific rhythm
Physical sensations & Movements	Pain, Touching of a specific body part, Moving a certain body part
Bodily Functions & Biological States	Urinating, Defecating, Sleep deprivation, Oversleeping, Waking up, Transition from waking to sleeping, Tooth brushing, Showering, Hunger, Fever, Menstruation
Physical & Emotional Stressors	Physical stress, Emotional stress
Miscellaneous	Others

#### Task Questionnaires

As mentioned in the experimental protocol, the overall perceived affective states were measured at the beginning and end of each lab session using the PANAS questionnaire, a widely used self-report questionnaire designed to measure two primary dimensions of mood: positive affect (PA) and negative affect (NA). The scale includes 10 items for positive emotions and expressions: *interested, excited, strong, enthusiastic, proud, alert, inspired, determined, attentive*, and *active*, and 10 items for negative emotions and expressions: *guilty, scared, hostile, upset, hostile, irritable, ashamed, nervous, jittery*, and *afraid*. The scoring for each of these items is provided on a five-point Likert scale: “*very slightly or not at all*”, “*a little*”, “*moderately*”, “*quite a bit*”, and “*extremely*”. The positive and negative affect score is calculated by adding individual Likert scale scores from the positive and negative items, respectively.

Additionally, the subjectively perceived workload, affect, and stress perceived for each task during the experiment were measured using the Likert scales for mental workload and stress, as well as the pairwise NASA-TLX questionnaire. The Likert scale showed five options to mark stress and mental workload levels: “*very very low*”, “*low*”, “*nor low nor high*”, “*high*”, and “*very very high*”. The pairwise NASA-TLX questionnaire was used to label the *mental demand, frustration, physical demand, temporal demand, performance*, and *effort* of a given task in six individual continuous scales ranging from 0 to 100. The pairwise comparison options were then presented to add weights to these continuous scales. The affective slider reports the level of arousal and valence of the participants on two continuous scales, each ranging from 0 to 1. The questionnaires were provided in German from validated sources, and available on GitHub. This multi-modal approach of contextualizing the physiological data with these behavioral measures enriches the dataset for a more comprehensive understanding of stress and workload dynamics in lab and situ settings.

### Data Processing

Physiological data were collected from two devices during the experiments. Hence, precise alignment of the data is required for meaningful multi-modal analysis. Instead of solely relying on the timestamps provided by the devices, which can often have non-synchronized clocks, different time zone settings, internal clock accuracies, or varying sampling delays, a physical shake-based synchronized protocol was utilized in this study using the Python package Jointly (https://jointly.readthedocs.io/en/latest/)^19^. The onboard acceleration sensors from Muse S and Empatica E4 recorded distinct motion peaks while the experimenter shook both devices vigorously for twelve seconds at the beginning and end of each session in the lab and in situ data collection. Jointly automatically detected these shake events across data streams, removed any temporal offsets, and applied time stretching to the data, aligning all signals precisely to a chosen reference sensor, which was the Empatica E4 in this case. Details of the synchronization protocol are available in the author’s previous publication on the data descriptor of healthy participants^10^.

The stretched data files from the **Raw** data are provided in the dataset inside the **Stretched** folder. The stretched lab data was chunked for specific tasks using the PsychoPy log files and the space bar tapping synchronization protocol and saved into the **Labeled** folder. The labeled data for the in situ setup was further extracted from the **Stretched** data based on timestamps. The synchronized and labeled sensor data is then preprocessed for further analysis.

Given the endless possibilities of preprocessing strategies for data from wearable EEG, PPG, EDA, and Temperature sensors^20–24^, a simple preprocessing pipeline was applied to the dataset as a clean and usable starting point for downstream analysis, which led to the **Preprocessed** folder in the data records. The raw blood volume pulse (BVP) data collected from the PPG sensors were first interpolated using linear and backfill methods to account for any missing or NaN values due to Bluetooth connections. A Savitzky-Golay filter with a window length of 41 and a polynomial order of 4 was used to smooth the signal by reducing high-frequency noise.

The EDA and the temperature signals used the same interpolation method since they were collected using the same device; connection issues would hamper all the sensors. Additionally, data loss could have happened due to the loose contact of the EDA sensors to the skin. Due to different wrist sizes and consequently infrequently loose electrode contacts, the app showed “device not on the wrist” whenever there were loose contacts.

The EEG signal also underwent linear interpolation methods for missing or Nan values. The high acceleration periods were identified from the acceleration signal, and corresponding EEG data were interpolated using the linear interpolation method. Furthermore, high-frequency noise was caused by the muscle activation of the scalp, and low-frequency disturbances (such as those occurring during heartbeats) were filtered using a band-pass filter (0.5-50 Hz). Since residual 50 Hz power line interference was empirically observed in some recordings, likely due to filter roll-off of the band-pass filter, an additional Notch filter was applied at 50 Hz. Although the frequency range exceeds the standard EEG frequency bands, this step enhances the quality of raw signals for potential future use in deep learning or time-series-based analyses. The EEG data were average referenced, extreme amplitudes beyond  ± 100*μ*V were clipped, and values exceeding this range were interpolated to reduce the impact of artifacts.

### Feature Extraction

While a wide range of features can be derived from the available data for each modality, and automated feature extraction tools (e.g., TSFRESH (https://tsfresh.readthedocs.io/), MNE-Python (https://mne.tools/stable/generated/mne.filter.filter_data.html), PyEEG (https://github.com/forrestbao/pyeeg), orNeuroKit2 (https://neuropsychology.github.io/NeuroKit/)) offer extensive options, the representative set of features computed in this work is commonly reported in the literature as relevant for assessing cognitive stress. Therefore, the preprocessed data was used to extract features from segmented time windows of 5, 10, 15, 20, 30, and 60 seconds and is saved as .pickle files in the **Features** folder inside data records. An 80% overlap was applied for each window size.

Time and frequency domain features were extracted from BVP data by using the Neurokit2 Python package, namely, mean (HRV-MeanNN), standard deviation (HRV-SDNN), and mean square of the RR intervals (HRV-RMSSD) as time domain features and normalized low (0.04 to 0.15 Hz) frequency power (HRV-LFn), high (0.15 to 0.4 Hz) frequency power (HRV-HFn) and the ratio (HRV-LFn/HRV-HFn) as frequency domain features. Frequency domain features were computed only for window sizes of above 30 seconds. Furthermore, the features extracted from preprocessed EDA signals capture both tonic and phasic components of the signal including the number of skin conductance response peaks (SCR-Peaks-N), the mean amplitude of the detected peaks (SCR-Peaks-Amplitude-Mean) reflecting short-term sympathetic arousal, the mean and standard deviation of the tonic EDA (slow-varying baseline) and the mean and standard deviation of the phasic EDA (rapid responses to stimuli) were computed.

Spectral, asymmetry and wavelet-based features were computed from the preprocessed EEG signal. From each of the four EEG channels across five standard frequency bands: *δ* (0.5–4 Hz), *θ* (4–7 Hz), *α* (8–12 Hz), *β* (12–30 Hz), and *γ* (30–50 Hz), power spectral density (PSD) was computed using Welch’s method with Hann window function. The average band power across the four channels (mean-*δ*, mean-*θ*, mean-*α*, mean-*β*, mean-*γ*) and the *θ*/*α* ratio were extracted as features. Asymmetry features were computed by subtracting the log-transformed spectral power of the right hemisphere from the left for each band (*δ*-asy, *θ*-asy, *α*-asy, *β*-asy, *γ*-asy). The frontal-*α*-asymmetry feature was computed using the *α* band power of the frontal electrodes (frontal *α*-asy). The power ratio features and engagement index were also computed from the band powers. Additionally, a 5-level discrete wavelet transform (DWT) was applied using the Daubechies-4 (db4) wavelet to decompose the EEG signal into different frequency bands using a recursive filter bank structure. While passing through high-pass and low-pass filters, detail coefficients (*dn*) and approximation coefficients (*an*) are produced, capturing high-frequency, transient components and low-frequency, slower oscillations, respectively. This decomposition into the wavelet coefficients a5, d5, d4, d3, d2, and d1 approximately aligns with known EEG frequency bands. Statistical features like mean, median, variance, standard deviation, skewness, kurtosis, root mean square (RMS), and signal energy were extracted from each channel’s coefficients. The name and description of the extracted features are listed in Table 6.

## Data Records

**Fig. 3:**
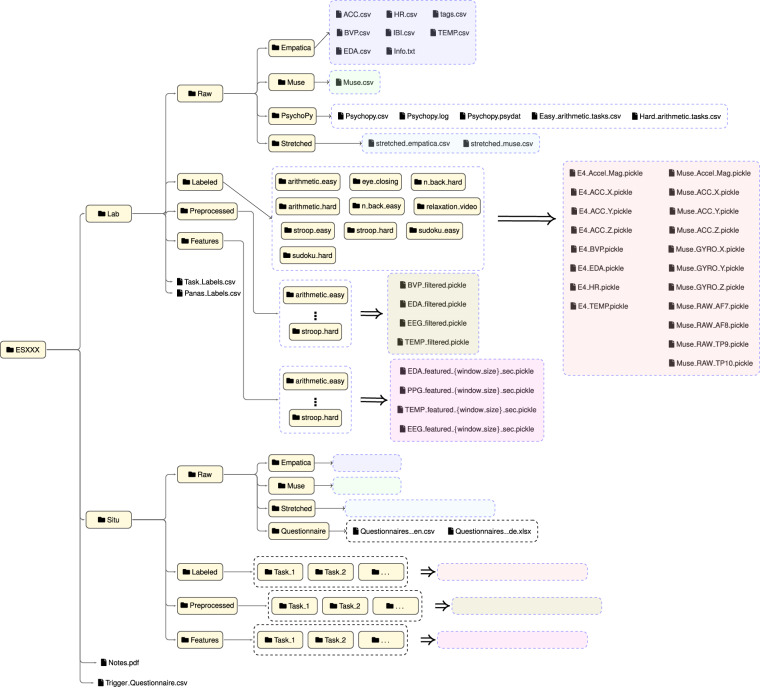
Overview of the folder structure of the data records. Example of one participant’s folder (**ESXXX**) is shown. The two top-level folders are **Lab**, which contains data from laboratory experiments, and **Situ**, which contains data from situational experiments. The two top-level files are “Notes.pdf”, which contains additional comments from the experimenter, and “Trigger_Questionnaire.csv”, which contains self-reported seizure triggers. Each top-level folder includes four sub-folders: **Raw** folder containing unprocessed sensor data from Muse and Empatica, Psychopy log files and Stretched data, **Labeled** folder containing synchronized and segmented raw sensor data, **Preprocessed** folder containing preprocessed labeled data, and **Features** containing extracted features from preprocessed data.

The complete dataset^25^ is available on Zenodo 10.5281/zenodo.16407549, run by the CERN Data Center. The dataset contains 20 topmost-level folders for each participant with the anonymous patient IDs: **ES304,** **ES491,** **ES474,** **ES472,** **ES452,** **ES415,** **ES446,** **ES437,** **ES404,** **ES375,** **ES362,** **ES313,** **ES260,** **ES257,** **ES247,** **ES208,** **ES228,** **ES141,** **ES140**, and **ES134**. Figure 3 depicts the example structure of one of these folders. The data is sub-grouped into Lab and Situ based on the experimental paradigm followed during data collection. As described in Table 5, the notes collected by the experimenter during both sessions are saved into the **Note.pdf** file, and answers to the seizure trigger questionnaire are recorded into an **Seizure_triggers.csv** file in the top-level folder for each participant. Both **Lab** and **Situ** folders are subdivided into four folders as follows:

- The **Raw** folder contains physiological data from **Muse** (in one .csv file) and **Empatica** (seven .csv files and one text file) devices as recorded, unprocessed, with potential artifacts present. The **PsychoPy** folder is present only for lab data, which logs the task timestamps and duration into a log and a CSV file, with additional .csv files containing the answers from arithmetic tasks when performed. Alternatively, the situ data has **Questionnaire** folder for task timestamp and task type, as described in Table 5. The **Stretched** folder contains the stretched data after using jointly during the data synchronization process (see section *Data Processing*).
- The **Labeled** folder holds the task-wise segmented data and is subdivided into the tasks performed in the lab and situ. Each task folder, i.e., **arithmetic_easy** or **Task_1**, comprises raw segmented data from Empatica E4 and Muse S in .pickle files. While the suffixes for the lab data segments, i.e., easy or hard, already define the designated task labels, the additional subjective scores are recorded in the **Task_Labels.csv** file with the corresponding folder names. On the contrary, the suffix from Situ data, i.e., Task_1, does not reflect the task difficulty but rather the chronology of the performed task. However, the subjective scores are depicted in the **Questionnaire_en.csv** file as described in Table 5.
- As mentioned in section *Data Processing*, the **Preprocessed** data is extracted after preprocessing the labeled data and saved into the respective folders.
- The **Features** folder holds extracted features from different *window_sizes* of 5, 10, 15, 20, 30, and 60 seconds and saved into the respective folders, only excluding the eye-closing sessions.

**Table 5:** Information and Example of the content of the descriptive files, i.e., ‘Notes.pdf’, ‘Trigger_Questionnaire.csv’, ‘Questionnaires__de.csv’ and ‘Questionnaires__en.csv’, present in different directory of the dataset as depicted in Fig. 3.

Filename Data Type	Information	Example
**Notes.pdf** Timestamp as *HH:MM*, followed by series of characters as free-text.	Denotes rough timestamps of performing tasks, disruption of Psychopy, Bluetooth disconnection, or recording interruption by the participants due to a question or feeling too tired to continue.	14:54 drinks water 14:54 Questionnaire n back (hard) 15:15 stopped recording to take a break.
**Trigger**_**Questionnaire.csv** CSV containing categorical responses as yes (“x”), maybe (“?”), or not applicable (“NA”) and ordinal values in each row representing an individual item.	Presents seizure-related factors in both English and German, each row associated with a unique item number and page reference. Answers to each factor as a provocative factor or an inhibitory factor are recorded as NA (not applicable), x (yes), or ? (maybe).	‘A specific memory’ (Seizure-related factor), ‘Eine bestimmte Erinnerung’ (Anfallsbezogene Faktoren (German)), ‘1’ (page), ‘1’ (item number), ‘NA’ (Anfall auslösend - provocative factor), ‘x’ (Anfall hemmend - inhibitory factor)
**Task**_**Labels.csv**	Provides the self-reported questionnaire—NASA-TLX, stress, mental workload, arousal and valence—scores for each task performed in the lab.	‘relaxation_video’ (Task), ‘5.0’ (Mental Demand), ..., ’0.673148’ (Arousal Score).
**Questionnaires**__**de.csv** CSV with integers, floating points, and series of characters in the columns, with each sample in an individual row.	Includes questionnaire number, timestamps, task details, and additional notes in CSV format. Text in the columns ‘task performed’ (‘Ich habe’), ‘break taken’ (‘Haben Sie während der Aufzeichnung Pausen gemacht?’), ‘additional information’ (‘Kommentar’) is provided as a combination of text and time stamps *HH:MM* by the participant. The pairwise NASA-TLX answers (in German) and the numeric scores of Likert scales of stress and mental effort were provided by the participants.	..., ‘1’ (questionnaire number), ‘XXX-XXX-XX’ (date), ‘14:13:00’ (questionnaire timestamp), ’13:26:00’ (recording started), ‘TV geschaut’ (task performed), ‘15’ (mental demand), ‘5’ (physical demand), ‘75’ (performance), ‘5’ (frustration), ‘Anstrengung’ (Effort vs Frustration) ‘3’ (mental effort level), ‘1’ (stress level).
**Questionnaires**__**en.csv** like Questionnaires__de.csv	like Questionnaires__de.csv Additional columns: ‘Tasks summary,’ ‘Note on feelings,’ ‘Task classification,’ ‘Short interruption,’ and ‘Note’ are transformed into English by the researchers with their interpretation. A short interruption is anything that interrupts the main task of the time block, e.g., a break or a toilet visit. ‘Note on feelings’ only if the participant provided information in writing. The pairwise NASA-TLX answers are translated into the original English version, and the weighted scores are calculated.	like Questionnaires__de.csv additionally ... ‘Watching Content on Smartphone’ (Task classification), ‘Short interruption’ (short interruption), ..., ‘57.5’ (Weighted Nasa Score).

**Table 6:** Extracted features from EEG, BVP, EDA, and Temperature modalities.

Modality	Feature name	Description
EEG	Power features	Mean PSD of *δ*, *θ*, *α*, *β*, *γ* across four channels
	Power ratios	*θ*/*α*, *α*/*θ*, (*θ* + *α*)/*β*, (*θ* + *α*)/(*α* + *β*), *θ*/*β*, *α*/*θ*
	Asymmetry	Asymmetry of *δ*, *θ*, *α*, frontal *α*, *β*, *γ*
	Engagement index	*β* * (*θ* + *α*)
	DWT features	Mean, median, variance, standard deviation, skewness, kurtosis, root mean square (RMS), and signal energy of each wavelet coefficients: a5, d5, d4, d3, d2, and d1
BVP	HRV-MeanNN	Mean of the RR intervals
	HRV-SDNN	Standard deviation the RR intervals
	HRV-RMSSD	Root mean square difference of successive RR interval
	HRV-LFn	Normalized low frequency (0.04 to 0.15 Hz) power
	HRV-HFn	Normalized high frequency (0.15 to 0.4 Hz) power
	HRV-LFn/HRV-HFn	Ratio between normalized low and high-frequency power
EDA	SCR-Peaks-N	Number of peaks of SCR
	SCR-Peaks-Amplitude-Mean	Mean amplitude of the SCR peak occurrences
	Tonic-EDA-Mean	Mean of tonic EDA
	Tonic-EDA-Std	Standard deviation of tonic EDA
	Phasic-EDA-Mean	Mean of phasic EDA
	Phasic-EDA-Std	Standard deviation of phasic EDA
TEMP	Mean-Temp	Mean of temperature values
	Std-Temp	Standard deviation of temperature values

*δ*, *θ*, *α*, *β*, and *γ* refer to the standard EEG frequency bands. DWT: discrete wavelet transform; HRV: heart rate variability, SCR: skin conductance response.

## Technical Validation

### Validation of study variables

#### Patient Characteristics

**Fig. 4:**
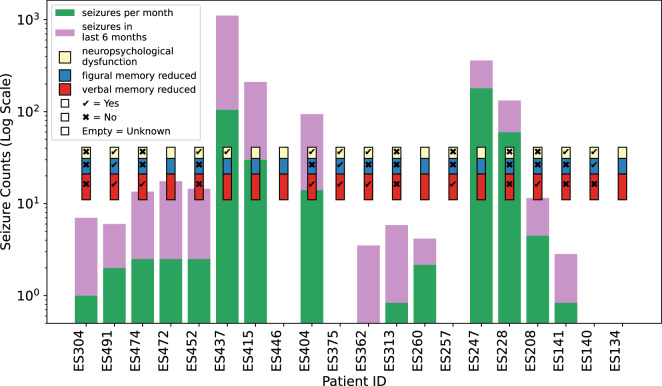
Patient IDs in x-axis with the bars of reported seizure frequency per month (in green) and in the last six months (in purple) in logarithmic scale in y-axis, the empty bars represent missing data. The yellow, blue and red boxes represent reduced verbal and figural memory and neurophysiological dysfunction, respectively, after conducting neurophysiological testing. *✓* and ✗ represents the agreement and disagreement of the respective category whereas empty value represents unknown or missing data.

Subsection *Patient Recruitment* illustrates the distribution of key patient characteristics, i.e., age, gender, and educational qualification. The age distribution captures both younger and older individuals diagnosed with epilepsy. The gender ratio within the dataset (8: 12) provides a proportionate diversity composition. The educational qualifications of the patients, ranging from foundational to advanced level, ensure that they can reliably engage with the study protocol of eliciting cognitive stress and self-reporting perceived stress. Additionally, a fundamental part of the dataset is the reported seizure frequency. The recruited patients had a mean seizure count of (20.4 ± 45.8) seizures with a maximum of 180 seizures per month. Figure 4 depicts the self-reported seizure frequency categorized by Loddenkemper *et al*.^26^ as daily (3 patients), persistent (12 patients)^27^ and Rare or Undefined category (5 patients). However, the classification can also be influenced by the phenomenon of under-reporting seizures^28^. This heterogeneity in seizure frequency is crucial for evaluating the correlation between seizure burden and cognitive stress across different patient profiles. Additionally, Fig. 4 shows the patients’ neurophysiological testing results: 35% and 15% of patients have reduced verbal and figural memory, respectively, and 40% of patients have neuro-psychological dysfunction. The neurological dysfunction for two patients was inferred from Lutz *et al*.^29^, whereas for the rest of the patients, explicit neuro-psychological evaluations have been performed. The combination of seizure frequency data and neurophysiological markers ensures a comprehensive dataset for analyzing the association of seizure risk with physiological and psychological variables. Therefore, the demographic and clinical characteristics of the patients closely reflect the intended framework of the experimental design.

#### Seizure Trigger Assessment

**Fig. 5:**
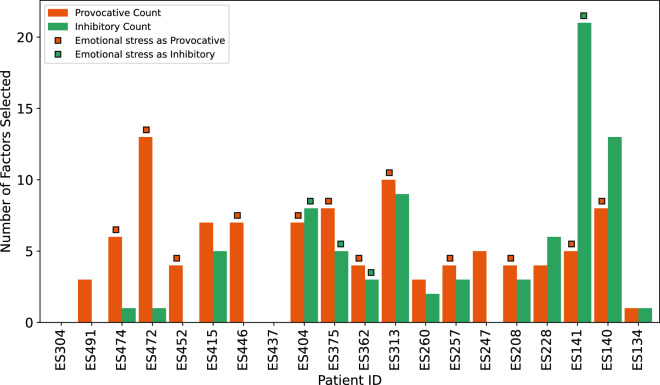
Patient IDs in x-axis with the bars of seizure provocative (in red) and inhibitory factors (in green) reported by the patients. All but one reported at least one provocative factor, 14 patients reported at least one inhibitor, empty value represents unknown or missing data. Emotional stress as provocative or inhibitory factor are marked with small rectangles of respective colors on top.

The analysis and results of the seizure trigger questionnaire are a core component of the dataset. Figure 5 showcases the variability in how individuals report their susceptibility to various environmental, emotional, and physiological factors. Excluding missing data from one patient, 94.7% patients reported experiencing at least one provocative factor, while 73.7% identified at least one inhibitory factor. Notably, one patient indicated no “known” triggers at all. The distribution of provocative factors is demonstrated in Fig. 5, where the three most frequently reported provocative factors are emotional stress (14 patients), sleep deprivation (10 patients), and physical stress (8 patients). Patients also reported additional provocative factors not listed in the provided list as special songs (“Faded” by Alan Walker), discontinuation of medication, stressful social interaction at work, i.e., working with unpleasant colleagues (but not in general stressful work situations that are unpleasant or stressful). Within the inhibitors illustrated in Fig. 5, the most mentioned factors were (avoiding) emotional stress (5 patients), sleep deprivation (4 patients), alcohol (4 patients), and physical stress (4 patients). Additional reported items not in the provided list were reducing exertion (e.g., playing only doubles, no singles), not being in the bathroom, avoiding unpleasant situations/persons or leaving place/conversation, and discontinuation of medication. These reported triggers align well with existing literature^3,18^, and provide additional items for future analysis. The consistent identification of emotional stress as both seizure-provocative and inhibitory factors strengthens the validity of the dataset and allows for correlation analysis between their self-reported trigger responses and corresponding physiological data.

#### Task Questionnaire Assessment

**Fig. 6:**
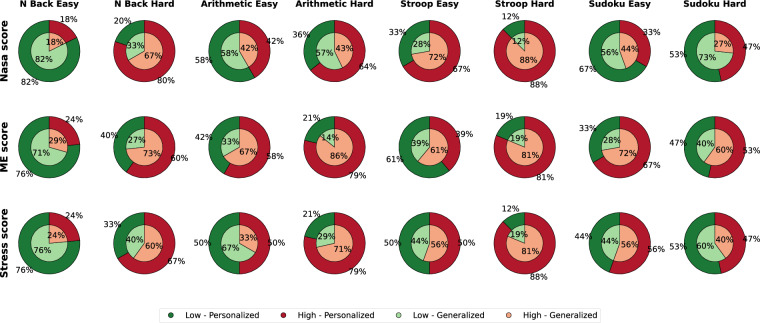
Subjective responses categorized into low and high task load groups based on two thresholding methods: the personalized threshold at the individual mean score across tasks (inner circles) and the generalized threshold at the overall median from all participants (outer circles). Green and light green represent lower workload levels, whereas red and light red represent higher workload levels. Task orders displayed in the bars are: N-back (easy  → hard), Arithmetic (easy  → hard), Stroop (easy  → hard) and Sudoku (easy  → hard). ME refers to Mental Effort.

The assessment of three scales (see Subsection *Behavioral Data Acquisition*) for participants’ perceived task demands ensures the robustness of the study design in eliciting the intended cognitive stress. Other than the defined task difficulties in the lab, the additional subjective responses reported after each task were further categorized into low and high task load groups based on two thresholding methods: the personalized threshold at the individual mean score across tasks and the generalized threshold at the overall median from all participants. The influence of the thresholding methods for the three scales is visible in Fig. 6, the inner and outer circles represent the personalized and generalized thresholds, respectively. The eight bars in the figure are organized left-to-right in four task pairs—N-back, Arithmetic, Stroop, and Sudoku—each displayed in its easy (green) condition first, followed by its hard (red) condition.

Most participants perceive the designed challenging tasks as difficult for the personalized threshold (Fig. 6 inner circle). However, the easy tasks are not always perceived as easy, i.e., the easy Stroop task was marked as a high load task by more than 67% and the easy arithmetic task was labeled as a high load task for more than 58% of the cohort. Therefore, a two-proportion z-test, also known as the test of proportions using the *proportions_ztest* function from the *statsmodels* (https://www.statsmodels.org/dev/generated/statsmodels.stats.proportion.proportions_ztest.html) library, was performed to statistically evaluate whether a significantly higher proportion of participants reported high workload levels in cognitively demanding tasks compared to non-demanding tasks. This test evaluates the null hypothesis that the proportion in demanding tasks is less than or equal to that in non-demanding tasks (*H*_0_: *p*_1_ ≤ *p*_2_), against the alternative that it is greater for the cognitively demanding tasks (*H*_1_: *p*_1_ > *p*_2_). To reject the null hypothesis, a critical z-value of  ±1.645 for a one-tailed z-test at *α* = 0.05 is required. The test yields z-values higher than the critical one and a statistically significant p-value (p < 0.005) for both NASA (*z* = 3.25, *p* = 0.00058) and stress (*z* = 2.74, *p* = 0.00306) scales, thereby rejecting the null hypothesis. In contrast, although the z-score for the mental effort scale (*z* = 2.38, *p* = 0.00861) falls above the critical value, the p-value (*p* < 0.005) suggests not rejecting the null hypothesis. Therefore, the analysis confirms that a significantly higher proportion of participants experienced high workload during cognitively demanding tasks for the two scales other than mental workload, validating the experimental manipulation while proposing further analysis of the robustness of the subjective scales.

For the generalized threshold (Fig. 6 outer circle)—while there is a trend toward a higher proportion of high workload reports in cognitively demanding tasks for Nasa (*z* = 1.25), stress (*z* = 2.16), and mental effort (*z* = 1.98) measures—statistical significance has not been found for any of the scales (*p* > 0.005). Therefore, the analysis confirms that the study successfully elicited a higher mental workload during demanding tasks as perceived by the participants individually. Furthermore, a Cohen’s Kappa analysis between thresholding methods provided moderate to substantial agreement (*κ* between 0.60 to 0.73) for all measures. The results suggest that the thresholding methods classify higher workload tasks similarly in many instances, and individual differences in workload perception lead to some inconsistencies.

**Fig. 7:**
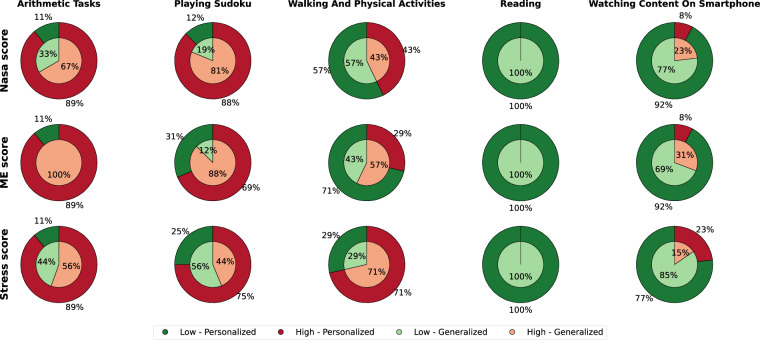
Subjective responses categorized into low and high task load groups based on two thresholding methods: the personalized threshold at the individual mean score across tasks (inner circles) and the generalized threshold at the overall median from all participants (outer circles). Green and light green represent lower workload levels, whereas red and light red represent higher workload levels. ME refers to Mental Effort.

Figure 7 depicts the subjective perception of cognitive stress for the self-chosen tasks in situ for personalized (inner circles) and generalized threshold (outer circles). Reading and watching content on a smartphone are primarily chosen as low-load tasks, and arithmetic and Sudoku are mostly chosen as high-load tasks for both thresholding methods. Walking and physical activities are perceived differently by participants and on different scales. Therefore, the intended study protocol has been achieved as the participants were instructed to choose balanced tasks between high and low workloads. Additionally, a Cohen’s Kappa analysis between thresholding methods shows substantial agreement for Nasa (*κ* = 0.71) and stress (*κ* = 0.66) scores but a moderate agreement for the mental workload (*κ* = 0.56) scale. Therefore, the individual differences in cognitive stress perception must be considered for further data analysis.

### Validation of physiological variables

**Fig. 8:**
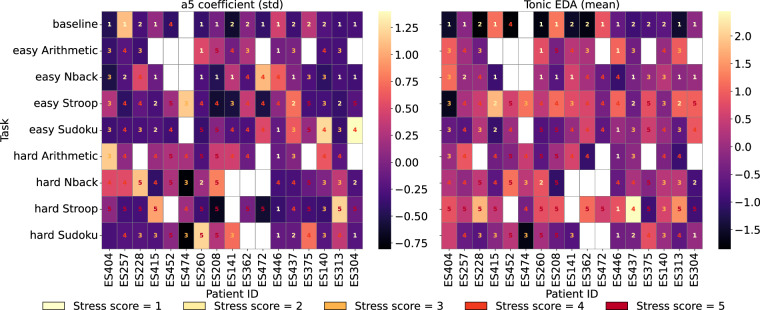
Participant-wise Z-scored mean *a*5 coefficient power standard deviation (left) and tonic EDA responses (on the right) for the tasks performed. The reported NASA-TLX scores are written inside the small boxes. The standardized values are displayed in fixed task order to enable comparability, resulting in missing values for some tasks (in white). The tasks were performed in randomized order. This visualization highlights individual variability in physiological variables for different cognitive stress responses.

In this section, a comparison of features from each modality extracted from 60-second windows is presented to assess the physiological sensitivity of the dataset to task-induced cognitive stress. The physiological responses measured by EEG and EDA sensors—*a*5 coefficient power (standard deviation) and tonic EDA features—in association with the task difficulty is depicted in Fig. 8. The figure displays participant-wise average Z-score distribution per task for (left) EEG *a*5 coefficient power (standard deviation), and the (right) tonic EDA feature presented in two heatmaps. The NASA-TLX difficulty scores are printed inside the heatmaps. The visual analysis indicates a trend of increasing *a*5 coefficient power (standard deviation) with task difficulty for both the defined difficulty level and NASA-TLX score. This suggests higher low-frequency brain activity as cognitive demands intensified; for instance, the baseline has lower power, mainly with a low NASA-TLX score, and the hard Stroop task shows a contrasting trend. Similar trend is visible for the tonic EDA feature, reflecting elevated sympathetic arousal in response to more demanding tasks. Supporting this visual observation, a linear mixed-effects analysis revealed that average *a*5 coefficient power (standard deviation) significantly increased with increasing task difficulty (*β* = 0.061, *p* = 0.036) when using the stress scale as a measure of difficulty. At the same time, tonic EDA significantly increased with task difficulty (*β* = 0.101, *p* = 0.045). The relatively small absolute magnitude of the coefficients reflects the use of normalization applied to the features. Nevertheless, these findings are consistent with established neurophysiological studies in the literature^30–32^.

Furthermore, the analysis was performed on task-only samples, i.e., without the baseline, and both coefficients remained positive; however, only the *a*5 coefficient power (standard deviation) continued to reach statistical significance. Therefore, the observed effects are strongest when baseline-to-task differences are included, while within-task difficulty differences produce a consistent but smaller trend.

**Fig. 9:**
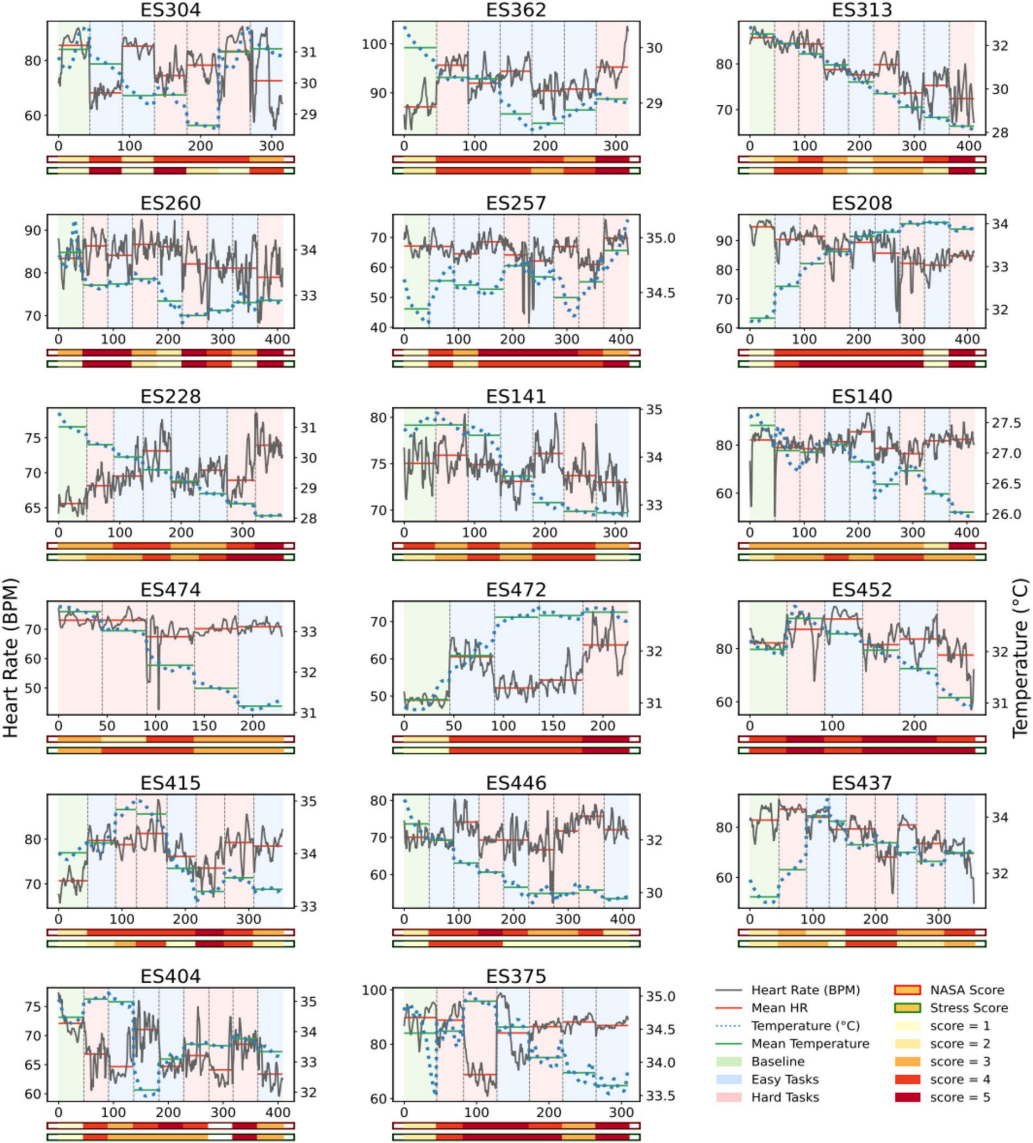
Experimental validity of heart rate fluctuations in beats per minute for each participant, e.g., **ES304**, while performing tasks in the lab. The baseline task is in green, and the defined low- and high-load tasks are marked in blue and red. The task names and the corresponding NASA score are also written inside the figures. The mean heart rate across each task is marked in red lines. The tasks are displayed in fixed order to enable comparability, but they are actually performed in randomized order.

Figure 9 illustrates the heart rate fluctuations in beats per minute and the skin temperature in Celsius with the corresponding subjective NASA-TLX and stress scores for each participant in each respective task. Based on the pre-defined task difficulty levels, the baseline relaxation tasks are marked in green, whereas cognitively low and high-demanding tasks are marked in blue and red, respectively. The task orders are printed according to the order of performance during the experiments, without accounting for any breaks taken or interruptions between the tasks.

While healthy individuals’ resting state heart rate ranges between 60 and 100, it varies significantly with the activity performed. Consequently, the figure reveals noticeable variations in heart rate as participants engage in different tasks, showing a trend of higher heart rates at the baseline for many participants. Further visual analysis of the plot indicates that it is difficult to see a common trend in heart rate variation concerning tasks performed by all participants. For instance, **ES362** shows a higher mean heart rate during high-demanding tasks and a lower during low-demanding tasks although the self-reported NASA-TLX scores show that easy tasks were mostly perceived as difficult tasks. Similar outcomes are visible for **ES472**. On the contrary, **ES228** follows a trend of increasing and decreasing heart rate with the perceived difficulty level (NASA-TLX score) of the tasks rather than the defined one. A paired t-test or Wilcoxon test chosen based on the normality of the data found no significant difference between pre-defined high and low-load tasks for heart rate feature. However, HRV-SDNN and HRV-RMSSD—both derived from heart rate features—remained statistically significant (*p* < 0.05) using the subjective stress scores, even after applying Benjamini-Hochberg correction, indicating a robust relationship between cardiovascular variability and perceived stress.

Furthermore, the skin temperature of a healthy individual is typically around 33 degrees Celsius, with changes during activity and room temperature. As mentioned, Fig. 9 shows these changes in skin temperature during the experiments, varying significantly for each participant. Since the room temperature was quite controlled during the laboratory data collection, the increased temperature for comparatively demanding tasks is visible for many participants, such as **ES362** and **ES472**. However, the paired t-test or Wilcoxon test chosen based on the normality of the data found no significant difference between pre-defined high and low-load tasks for temperature features. In contrast, statistical significance is found in the standard deviation of temperature when the data is divided according to the stress score (*p* < 0.05); this effect did not remain significant after multiple-comparison correction.

Consequently, at least one representative feature from the PPG (e.g., HRV-SDNN, HRV-RMSSD) and temperature (e.g., standard deviation) modalities showed statistically significant separability—only the PPG features remained significant after correction—when cognitive stress was defined based on subjective scores. In addition, representative features from EEG (e.g., *a*5 coefficient power (standard deviation) and EDA (e.g., tonic level) modalities demonstrated significant directional associations with subjective scores informed by prior literature. Therefore, the physiological responses captured in the dataset suggest a difference in perceived cognitive stress with defined task difficulty, indicating good construct validity. The significant physiological features in response to subjectively experienced load support the convergent validity of the dataset with established psychological instruments, and the different cognitive stress measuring techniques—subjectively (via ratings) and objectively (via physiological signals)— point in the same direction. However, while additional features may also show discriminative potential, the current results are sufficient to support the validity and suitability of the proposed dataset for cognitive stress detection.

## Usage Notes

### Usage of Data Acquisition Interface

The data acquisition interface, PsychoPy, can be reused for cognitive stress elicitation experiments with the synchronization protocol included. The timestamp logging, task-duration settings, and responses to the tasks are automated. The experiments can be reduced by selecting fewer tasks or reducing duration; on the contrary, the experiments can be extended by incorporating new tasks and existing task durations. The interface is capable of recording data in two languages, English and German, with the possibility of incorporating more languages into the interface.

### Usage of Dataset

The several components of the proposed dataset can be used for multiple research purposes. The raw physiological data retaining full signal information can be used for unsupervised or self-supervised deep learning models, signal quality assessments of low-cost wearable sensors, and pretraining neural networks. The labeled data with predefined and self-reported cognitive stress labels are essential for supervised learning and regression or classification purposes of stress detection models. The labeled data is furthermore suitable for applying comprehensive pre-processing techniques. The minimally pre-processed data offer extensive feature exploration from individual modalities. The features provided with the dataset can be used as input for machine learning models and are useful for multi-modal fusion modeling. The patient-specific seizure trigger information facilitates clustering based on trigger responses. Personalized seizure risk influenced by stress can be modeled through multi-modal approaches that integrate extracted features, seizure trigger information, and subjective stress reports. Moreover, the dataset is also suitable for a patient vs control analysis for a direct statistical comparison of disease-specific versus normative responses and can also serve as a primary source domain for a transfer-learning pipeline on publicly available seizure forecasting or prediction datasets.

## Data Availability

The dataset presented in this Data Descriptor is available through the Zenodo repository at 10.5281/zenodo.16407549. The repository includes multi-modal physiological recordings (EEG, EDA, PPG, and temperature) and accompanying metadata collected from epilepsy patients during cognitive stress experiments, both in controlled laboratory and in-situ sessions. As described in the Data Records, the repository is organized into four directories for each participant: i) **Raw**: continuous, synchronized recordings from each device in their native sampling rates and formats, ii) **Labeled**: task-wise segmented raw data, iii) **Preprocessed**: cleaned data with standard preprocessing across modalities, iv) **Features**: extracted physiological and statistical features with different window sizes. The folders contain files in either *.csv* or *.pickle* format. Additional files in the repository contain “Notes”, which include any information noted during or after the experiments, and “Questionnaires”, which report seizure-related factors and task difficulty levels. The dataset is well documented in this current data descriptor and in the Zenodo repository. The detail on data loading is made available at https://github.com/HPI-CH/EPIStress.

## Code availability

**Data Acquisition Interface.**

The Python code of the data acquisition platform, PsychoPy, can be found at https://github.com/HPI-CH/EPIStress/tree/main/Psychopy. The repository is self-explanatory with a detailed description in the README file.

**Data Loading.**

The Python code to load the raw data is available at https://github.com/HPI-CH/EPIStress. The repository contains Python codes for synchronizing, labeling and preprocessing the raw data and extracting features from the preprocessed data. The repository is self-explanatory, with a detailed description in the README file.
